# Clarification of the Antagonistic Effect of the Lipopeptides Produced by *Bacillus amyloliquefaciens* BPD1 against *Pyricularia oryzae* via In Situ MALDI-TOF IMS Analysis

**DOI:** 10.3390/molecules21121670

**Published:** 2016-12-03

**Authors:** Jen-Hung Liao, Pi-Yu Chen, Yu-Liang Yang, Shu-Chen Kan, Feng-Chia Hsieh, Yung-Chang Liu

**Affiliations:** 1 Department of Chemical Engineering, National Chung Hsing University, Taichung 40227, Taiwan; colvin@taifer.com.tw (J.-H.L.); sherrykan0402@gmail.com (S.-C.K.); 2 Biopesticides Division, Taiwan Agricultural Chemicals and Toxic Substances Research Institute, Council of Agriculture, Taichung 41358, Taiwan; 3 Agricultural Biotechnology Research Center, Academia Sinica, Taipei 11529, Taiwan; jasper28@gate.sinica.edu.tw (P.-Y.C.); ylyang@gate.sinica.edu.tw (Y.-L.Y.)

**Keywords:** *Bacillus amyloliquefaciens* BPD1, lipopeptides, fengycin, MALDI-TOF IMS, SEM

## Abstract

This study tried to clarify the antagonistic effect of the lipopeptides secreted by *Bacillus amyloliquefaciens* strain BPD1 (Ba-BPD1) against *Pyricularia oryzae* Cavara (PO). To determine the major antifungal lipopeptides effective against PO, single and dual cultures were carried out in solid-state media. The matrix-assisted laser desorption/ionization–time of flight imaging mass spectrometry (MALDI-TOF IMS) was used to identify the most effective lipopeptide in situ. Meanwhile, the morphology of pathogen fungi treated with lipopeptides was observed via the SEM. Of the three lipopeptide families, surfactin, iturin, and fengycin, the last was identified as the most effective for inhibiting mycelium growth and conidial germination of PO. The conidia and hyphae of fengycin-treated PO were shown to become deformed and tumorous under exposure. This study provides insights into the antagonistic effect of Ba-BPD1 against fungal phytopathogens. Such insights are helpful in the development of reagents for biological control applications.

## 1. Introduction

Consumed by more than 50% of the global population, rice is one of the world’s most important crops [1]. Rice blast disease, caused by *Pyricularia oryzae* Cavara (PO), is one of the most destructive and widely spread diseases [2], resulting in serious yield losses in countries such as India, the Philippines, and Nigeria [3,4,5]. In Brazil, rice blast is considered to be the major yield constraint factor [6]. Several methods, such as the use of fungicides, resistant cultivars, and biotechnological approaches, have been employed to control the phytopathogen diseases [7]. Chemical fungicides are the most widely used in-field application; however, they are hazardous to the ecosystem [8]. In contrast, biological approaches through the use of microbial antagonists are considered to be more environmentally friendly, effective, and sustainable in an agricultural setting. Several microorganisms, such as *Bacillus* spp. and *Pseudomonas* spp., have been reported to be potential strains for rice blast disease control [9,10].

*Bacillus amyloliquefaciens* has been proposed for its agricultural disease control properties [11]. Its ability to produce various antifungal lipopeptides has been considered to be the key factor in controlling phytopathogen diseases [11,12]. Most of these lipopeptides have a molecular weight of around 1000–1600 Da and are synthesized non-ribosomally via a multi-enzyme biosynthesis pathway [12,13]. The representative lipopeptides are classified into three families: surfactin, iturin (mycosubtilin, iturin A, and bacillomycin), and fengycin [14]. All were reported to possess respective antifungal activities for inhibiting the growth of filamentous fungi [15,16,17]. For instance, studies indicated that surfactin had strong synergistic functions when applied in combination with iturin A [18] or fengycin [19]. Arrebola et al. proposed that iturin A was the major antagonistic lipopeptide [20]; reports concerning the antimicrobial effect of fengycin are still few compared to reports on iturin.

Pathak et al. have isolated the fengycin lipopeptide family from the antagonistic strains of *B. subtilis* [21]; however, no direct evidence of using fengycin as a standard for inhibiting pathogenic fungi has yet been provided. In addition, none of the reports have studied the effect of lipopeptide secretion during in situ antagonistic testing, and little has been done to assess the effect of individual lipopeptides on phytopathogen.

In this study, a strain of Ba-BPD1 with a wide spectrum of antimicrobial capacities was isolated from local mountain soils. This strain exhibits high antagonistic activity against a large number of plant pathogens, particularly against PO that causes rice blast disease. Arguelles-Arias et al. indicated that *Bacillus amyloliquefaciens* has potent antibiotics and other secondary lipopeptides for biocontrol of plant pathogens. In this study, three families of lipopeptides, i.e., iturin, fengycin, and surfactin, were found in the cultivation of strain Ba-BPD1. The antagonistic effect of using lipopeptides to inhibit PO growth was detected. The lipopeptides produced within the interspecies competition zone were tracked in situ via matrix-assisted laser desorption/ionization imaging mass spectrometry (MALDI-IMS), which is capable of visualizing the spatial distribution of multiple compounds from the microbial growth on agar media [22,23,24]. Scanning electron microscopy (SEM) observation of the morphology of PO under antagonistic testing was also carried out. The antagonistic effect of the lipopeptides produced by Ba-BPD1 against PO was illustrated and clarified.

## 2. Results and Discussion

### 2.1. Identification of the Strain Ba-BPD1

**Figure 1:**
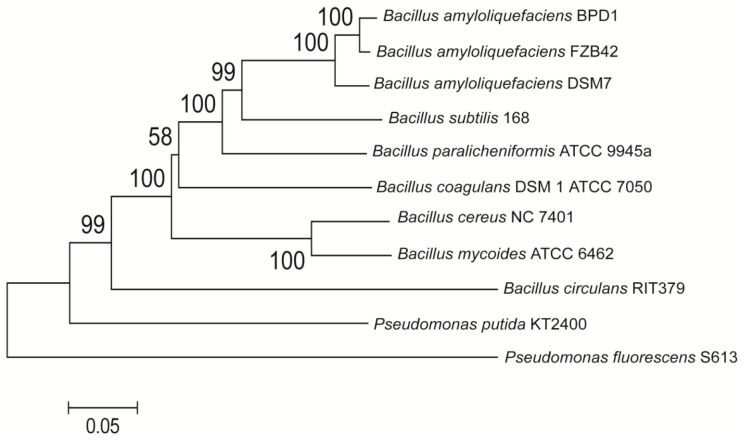
Neighbor-joining phylogenetic tree analysis of *Bacillus* spp. based on gyrA nucleotide sequences (2246 bp) using *Pseudomonas* spp. as an outgroup. The evolutionary distances were calculated by the p-distance method based on 1000 bootstrap replication. The phylogenetic tree was computed using the *MEGA6.0* program. (Molecular Evolutionary Genetics Analysis, a free software obtained from http://www.megasoftware.net/).

A strain isolated from Taiwan mountain soils was found to possess a wide spectrum of antimicrobial capacities for inhibiting fungal or bacterial diseases. The results of the antagonistic spectrum were listed as the Supplementary materials for reference. This strain was identified as *Bacillus amyloliquefaciens* via the gyrA rDNA sequence (GenBank accession number: KX819300) and was named Ba-BPD1. The neighbor-joining phylogenetic tree in Figure 1 shows the similarities within this strain.

### 2.2. Identification of the Lipopeptides of Ba-BPD1

**Figure 2:**
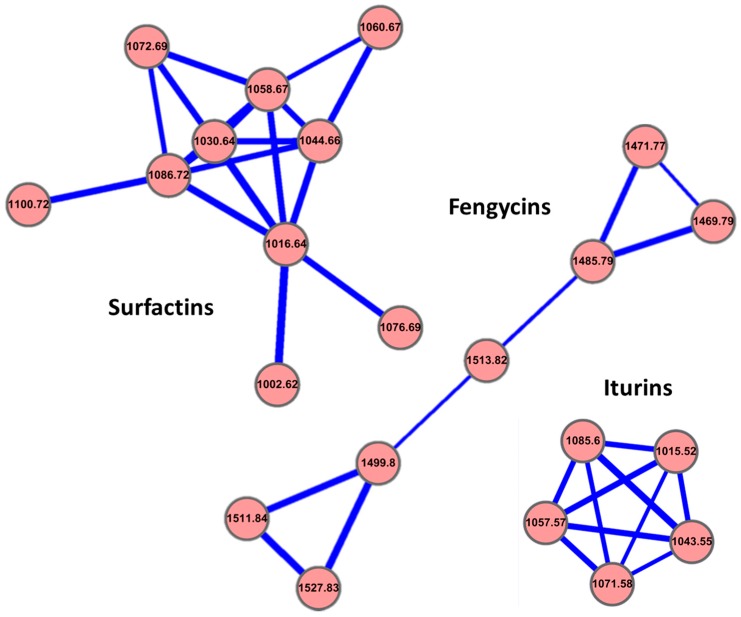
Molecular networking of lipopeptide family clusters produced by Ba-BPD1 in LB medium. The lipopeptides were grouped into three clusters: surfactins, iturins, and fengycins. Values of *m/z* [M + Na]^+^ were used to present each group of lipopeptides.

Strain Ba-BPD1 was cultivated in Luria–Bertani (LB) medium. The fermentation broth was then harvested and the metabolites were analyzed. The molecular networking analysis based on LC-MS/MS data demonstrated that Ba-BPD1 produced three distinguishable groups of lipopeptides [25,26]. Surfactins were the first group, including *m/z* of 980.6276, 994.6445, 1008.6606, 1022.6745, 1036.6905, 1038.6667, 1050.7073, 1054.7013, 1064.7214, and 1078.7374. Iturins were the second group, including *m/z* of 1015.5179, 1043.5489, 1057.5686, 1071.5805, and 1085.5958. Fengycins were the third group, including *m/z* of 1435.7688, 1447.8038, 1449.7848, 1463.8012, 1477.8159, 1489.8540, 1491.8308, and 1505.8460 (Table 1). Within the same lipopeptide family, the *m/z* differences of 14 suggested a series of homologous molecules having different lengths of fatty acid chains (i.e., CH_2_ = 14). Therefore, the mass shifts of 14 were considered to occur within the same non-ribosomal lipopeptide family with different fatty acid chain lengths by methylation. In addition, the *m/z* differences of 22 in the MS spectrum were expected to be the same molecule as for the protonation [M + H]^+^ or the sodium ion form [M + Na]^+^. The standard lipopeptides (marked with # in Table 1) were used to check the composition similarity for the lipopeptides produced in the Ba-BPD1 LMS fermentation broth. To systematically correlate the relationship of these lipopeptides produced in the broth by this strain, the software Cytoscape was used to generate the molecular networking of the lipopeptide family clusters, as visualized in Figure 2.

### 2.3. MALDI-TOF IMS Analysis

**Figure 3:**
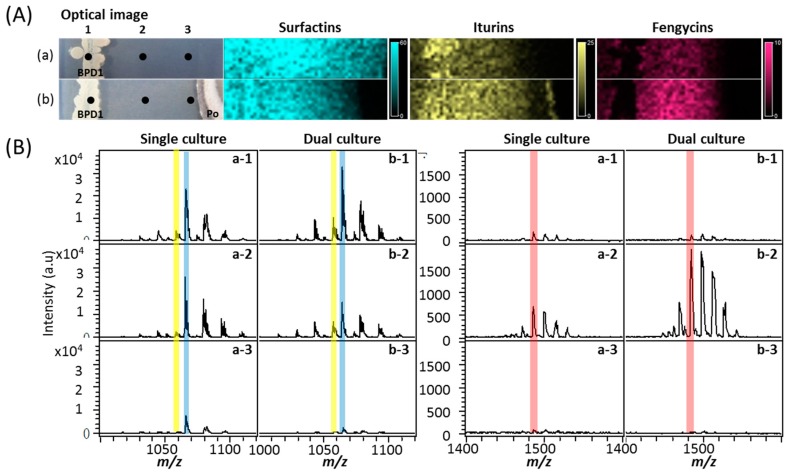
(**A**) MALDI-TOF IMS profiles of Ba-BPD1 (BPD1) in (**a**) single culture with *P. oryzae* (Po) and (**b**) dual culture. Lipopeptides (surfactin, iturin, and fengycin) were detected in different zones, 1–3, on the agar plate, respectively; (**B**) The mass spectra of compounds detected in zones 1–3 are shown.

To test the antagonistic effect of Ba-BPD1 against PO, both a single culture and a dual culture were set up. PO was clearly growth-inhibited by Ba-BPD1 in the dual culture (See Section 3.4 and Figure 3). To determine the major antifungal lipopeptides produced by Ba-BPD1, the spatial distribution of the lipopeptides in the inhibition zone was evaluated in situ via the MALDI-TOF IMS. Results indicated that three lipopeptides, i.e., iturin, fengycin, and surfactin, were produced by Ba-BPD1. In the MALDI-TOF analysis in Figure 3A, surfactin was marked with blue; iturin was marked with yellow and fengycin was marked with red. In addition, to identify the lipopeptides’ spatial distribution on the plates, Ba-BPD1 colonies were labeled as zones (a)-1 and (b)-1, and the inhibition zones were labeled as (a)-2 and (b)-2 for the single and dual cultures, respectively; the PO colony was labeled as position (b)-3 in dual culture and the same location was marked as (a)-3 in the single culture.

As shown in Figure 3A, it was found that in the single culture, surfactin was produced uniformly and wholly around zones (a)-1 to (a)-3, and iturin was observed mainly in zone (a)-1, where Ba-BPD1 cells existed; however, only a small amount of fengycin was found in zone (a)-1. In contrast, in the dual culture, surfactin was decreased clearly at zone (b)-3, where the PO strain grew. This indicated that some of the metabolic pathways for surfactin production might have shifted to iturin and fengycin, or the surfactin close to PO was decomposed or degraded by the PO strain. In the antagonistic dual culture, iturin was increased at zones (b)-1 and (b)-2, whereas fengycin was dramatically enhanced mainly at zone (b)-2 (the inhibition zone). This suggests that surfactin is spontaneously secreted, whereas most fengycin and iturin are produced when Ba-BPD1 meets the competition stress from pathogen PO.

To further confirm the observation, mass-spectra were provided in Figure 3B. The *m/z* peak value of 1064 was used to represent surfactin; *m/z* of 1057 for iturin and *m/z* of 1485 for fengycin. In the spectra, surfactin was decreased at zones (b)-2 and (b)-3 in the dual culture plate. This suggests that surfactin diffusion was inhibited or surfactin was degraded by PO during dual culture. Hoefler et al. proposed that microbial antagonists might secrete metabolites against the growth of competing species. The competitors might also develop resistance to the antagonistic metabolites [27]. Additionally, in dual culture, iturin increased slightly in zones (b)-1 and (b)-2, and fengycin increased three-fold in zone (b)-2 compared to in zone (a)-2 (single culture). This indicates that both iturin and fengycin might be inducible defense metabolites of Ba-BPD1 against PO. This observation is consistent with that reported by Liu et al. that iturin and fengycin exerted synergistic functions on pathogen inhibition [15]. It is of interest that the marked increase of fengycin in dual culture might imply that fengycin is the most critical antagonistic metabolite secreted by Ba-BPD1 under external stimulation.

### 2.4. Fermentation Medium Tested for Fengycin Production

**Figure 4:**
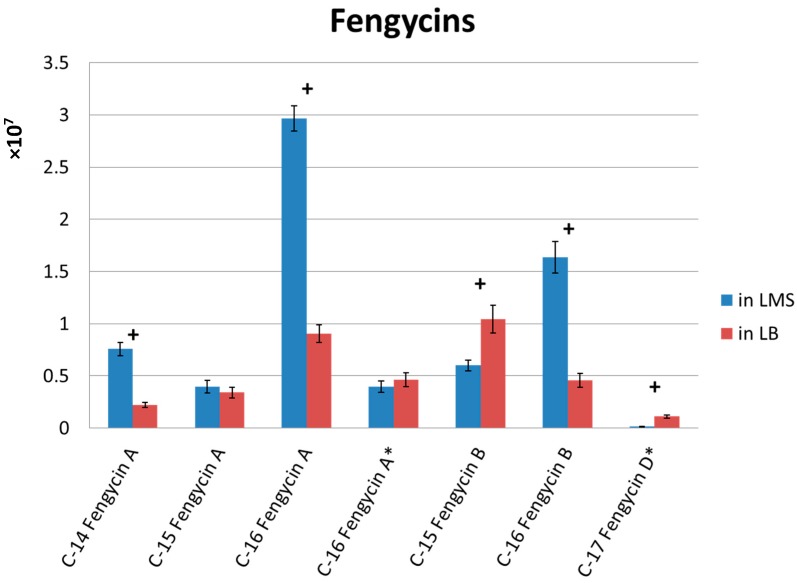
Quantification of fengycins in Ba-BPD1 cultured in LMS and LB media. * Linear form of lipopeptides; + significant difference with a *p*-value < 0.005 (all data were analyzed by two sample *t*-tests). Each experiment was conducted three times and expressed as the mean ± standard deviation.

Although LB medium is a common medium for *Bacillus* sp. cultivation, it is still unsuitable for industrial use due to cost concerns. To design a medium fit for industrial scale-up use, a complex medium LMS (10 g/L lactose, 5 g/L molasses, and 20 g/L soy protein) was chosen after the preliminary tests. To understand the effect by the use of LMS medium, the two media, LB and LMS, were used to test the production of fengycin by Ba-BPD1. The harvested broths were collected and analyzed by LC-MS. Figure 4 shows the results. The levels of C-14 fengycin A and C-16 fengycins A and B in LMS broth were almost twice those in LB broth. In addition, the use of LMS can produce similar lipopeptide metabolites as are produced by using LB. Since fengycin is the key compound in the broth, the marked enhancement of fengycin in LMS medium means it is a good substitute for LB medium.

### 2.5. Antifungal Bioassay and SEM Observations

**Figure 5:**
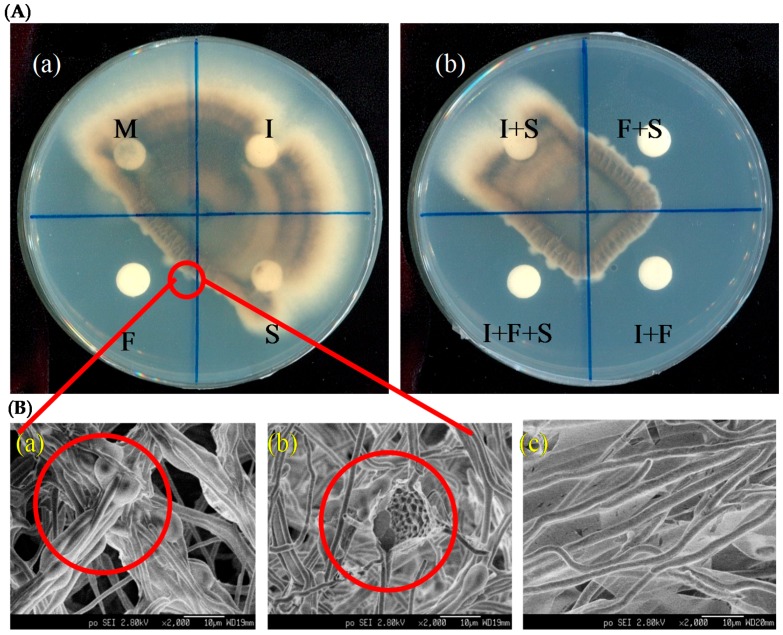
(**A**) Antifungal assay of commercial lipopeptide standards against *P. oryzae*, where I: iturins, F: fengycins, S: surfactins, and M: methanol; (**B**) The morphology for *P. oryzae* hyphae collected at the inhibition border via SEM observation. The fengycins-treated *P. oryzae* hyphae were (**a**) swollen or (**b**) cracked, where (**c**) was the untreated hyphae.

In order to find the major lipopeptide attributed to the antagonistic effect against PO, all the lipopeptide standards were used in the preparation of antagonistic plate tests. Figure 5 shows the results. Fengycin standard revealed a significant inhibitory effect on hyphal growth of PO; however, as shown in Figure 5A-a, treatment with surfactin and iturin standards did not show any inhibitory effect. In addition, the combination tests in Figure 5A-b showed that all combinations with fengycin gave biocontrol activity against PO. Based on the above observation, it is evident that fengycin is the major lipopeptide effective against PO in this study.

In the test using fengycin treatment, the hyphae at the antagonistic border were observed as being burned or corroded. To study more clearly the hyphal morphology at the demarcation line, SEM was applied; Figure 5B shows the results. SEM observation displayed that the hyphal morphologies of PO around the demarcation line were deformed, i.e., swollen (Figure 5B-a) or even cracked (Figure 5B-b). This observation is consistent with that reported by Tang et al. concerning the effects of fengycin on morphological damages in fungal pathogens [28]. In contrast, the untreated hyphae were even and uniform (Figure 5B-c). In addition, globular hyphae were found on the fengycin-treated plate but were rarely seen on the untreated one. These results further indicate that fengycin is likely the major compound for PO inhibition. The findings mentioned above provided understanding of the function of fengycin in the antagonism of Ba-BPD1 against PO, which might be helpful in rice blast biocontrol.

### 2.6. The Effect of Ba-BPD1 Broth on the Germination of Conidia of P. oryzae 

**Figure 6:**
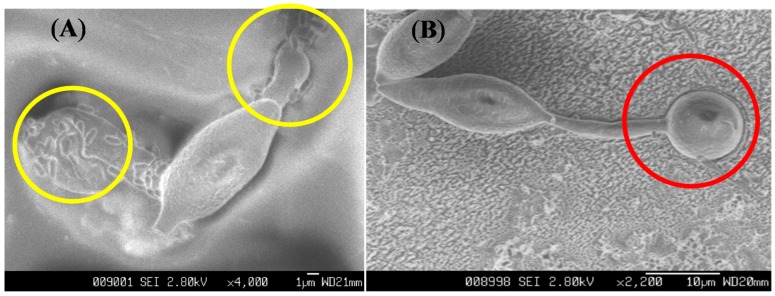
When the deformed germ tubes were treated with Ba-BPD1 broth, no appressoria was observed (**A**). The appressoria was formed at the tips of the germ tube when conidia of *P. oryzae* germinated on the host (**B**).

To test the inhibition effect of Ba-PBD1 broth on the germination of PO conidia, the procedure described in Section 3.6 was followed. SEM was used to observe the change in morphology. As shown in Figure 6a, no germination was observed when PO was treated with LMS broth, whereas the untreated PO’s growth was unlimited (Figure 6b). It is of interest that the germ tube (marked as the yellow circle in Figure 6a) of PO conidia was deformed, swollen, and unable to form appressoria (marked as the red circle in Figure 6b) when treated with Ba-BPD1 broth (1.25 × 10^8^ CFU/mL). This revealed that the compounds in LMS broth, especially fengycin, can inhibit conidia germination via deforming the conidia and germ tube of PO. Furthermore, the formation of fungi appressoria can also be stopped, which might prevent fungal uptake of essential nutrients from plants [29].

## 3. Experimental Section

### 3.1. Chemicals

Lipopeptide standards, i.e., iturin A (CAS No. 52229-90-0), surfactin (CAS No. 24730-31-2) and fengycin (CAS No. 102577-03-7), were purchased from Sigma-Aldrich Co. (St. Louis, MO, USA). Luria–Bertani medium was purchased from Sigma Chemical Co. All other chemicals were of analytical grade and were purchased from the local dealer.

### 3.2. Strain and Culture Conditions

The strain Ba-BPD1 was isolated from the soil in Lishan, Taichung County, Taiwan. Then it was incubated, identified, and deposited at the Bioresource Collection and Research Center in Taiwan (BCRC 910395) and the Leibniz-institut DSMZ-Deutsche Sammlung von Mikroorganismen und Zellkulturen GmbH in Germany (DSM 21836). A patent was also applied in the USA (US 2010/0143316A1). Ba-BPD1 was cultured on Luria–Bertani (LB) medium at 30 °C for 16 h, followed by centrifugation to collect the cells’ pellet and then mixing with fresh LB broth plus 20% glycerol. It was then deposited at −80 °C as the stock. For the LB and LMS (10 g/L lactose, 5 g/L molasses and 20 g/L soy protein) fermentation, 1 mL of Ba-BPD1 stock was inoculated to 60 mL LB and LMS medium, respectively, and then cultured at 30 °C, at 200 rpm for 72 h. Then the fermentation broth was collected for the LC-MS analysis. LMS medium was used for lipopeptide production, MALDI-TOF IMS analysis, and conidial morphology observation experiments.

### 3.3. LC-MS and LC-MS/MS Analysis

For the LC-MS analysis, Ba-BPD1 was inoculated in LB and LMS broth at 30 °C for 72 h and then extracted by either ethyl acetate or *n*-butanol. The extracts were concentrated to dryness by rotary evaporation at 45 °C and then dissolved in methanol for LC-MS analysis. A C18 column (ACQUITY UPLC BEH-C18, 130 Å, 1.7 μm, 2.1 × 100 mm) was used with the following gradients: 0–6 min at 5%–99.5% of acetonitrile (ACN), 6–8 min at 100% of ACN, 8–8.2 min at 99.5%–5% of ACN and 8.2–10 min at 5% of ACN. The flow rate was set at 0.4 mL/min. The mass data were acquired in triplicate using the Thermo Orbitrap Elite system. Mass data were acquired in profile mode and positive mode, with mass range *m/z* 100–1500 with a resolution of 30,000 at *m/z* 400. For tandem mass data, the top five intense ions from each full mass scan were selected for collision-induced dissociation (CID) fragmentation. For CID, the isolation width was 2 *m/z* and the selected ions were fragmented with a normalized collision energy of 30.0 or 35.0 eV, activation Q 0.250 ms, activation time 10.0 ms, and a resolution of 15,000 at *m/z* 400. The mass data were converted to mzXML file formats followed by applying to software GnPS (https://gnps.ucsd.edu/ProteoSAFe/static/gnps-splash.jsp) to generate molecular networking; data were then visualized in Cytoscape (Institute of Systems Biology, Seattle, WA, USA).

### 3.4. MALDI-TOF IMS Analysis

For the MALDI-TOF IMS analysis, the PDA agar plate was smeared with a streak of Ba-BPD1 from LMS broth, 2 cm from the plate edge. Meanwhile, a 1 cm diameter agar plug of PO was inoculated onto the same plate. The distance between Ba-BPD1 and PO inoculum was 5 cm. The plates were incubated at 30 °C for 12 days and the regions of interest containing side-by-side inoculated microorganisms or singly grown microorganisms were cut and placed on an indium tin-oxide-coated glass slide. All slides with target samples were then covered with a thin layer of universal MALDI matrix using the sieve method and were dehydrated at 37 °C to generate a uniform distribution of crystalline matrix. The sample slides were subjected to a Bruker Autoflex Speed MALDI-TOF/TOF MS for collecting IMS data with a typical mass range of 100–2000 Da. The IMS data were analyzed using Bruker FlexImaging 3.0 software (Bruker, Bremen, Germany).

### 3.5. Antifungal Bioassay

The antifungal activity of lipopeptide standard agents toward PO was tested on PDA plates. Some 30 μL of 0.25% standard agents, iturin A (consisting of at least six iturin A isomers), fengycin, and surfactin, were loaded on an 8-mm paper disc and placed 2 cm from the edge of the plates. An agar plug of PO (about 1 cm in diameter) cut from the leading edge of the culture (grown on PDA at 25 °C for five days) was simultaneously placed in the center of the antagonistic plate. The plates were incubated at 25 °C for 12 days. A slice of sample agar was cut from the antagonistic plate and stuck on the specimen holder, followed by freezing using liquid nitrogen. Then the specimen was transported to the vacuumed chamber via a specimen exchange rod. The hyphae of PO at the border line were examined under the field emission scanning electron microscope (JEOL JSM-6330F, JEOL Ltd., Tokyo, Japan). 

### 3.6. Observation of Conidial Morphology

The LMS broth of Ba-BPD1 was also tested for the effect on conidia. Conidia of PO were used as targets, and the concentration of conidial suspension was adjusted to 5 × 10^5^ conidial/mL. In each test, 25 μL of 20-fold diluted LMS broth of Ba-BPD1 (stock concentration of 5 × 10^9^ CFU/mL) was mixed with 25 μL of conidial suspension and incubated at 25 °C for 12 h. The conidial morphology was observed under the field emission scanning electron microscope (JEOL, JSM-6330F).

## 4. Conclusions

In this study, a novel strain Ba-BPD1 was isolated and tested for its antagonistic activity. SEM observation and MALDI-TOF IMS analysis were adopted to clarify the effect of the major antagonistic lipopeptides. The MALDI-TOF IMS analysis indicated that fengycin was inducible and distributed mainly in the inhibition zone in dual culture, whereas iturin and surfactin were distributed in both the cell and inhibition zones in single culture. The use of standard lipopeptides showed that fengycin was a key compound secreted by Ba-BPD1 and was responsible for the antifungal effect toward PO. In addition, via SEM observation, fengycin could cause the deformation of hyphae and conidia of PO. In this study, it was evident that fengycin acted as the major antagonistic compound toward PO. This understanding might help in the preparation of biocontrol reagents for the rice blast disease control.

## Figures and Tables

**Table 1:** Lipopeptide composition of Ba-BPD1 analyzed by LC-ESI MS.

Compounds	Molecular Formula	t_R_ (min)	[M + H]^+^	[M + Na]^+^	Observed [M + H]^+^	Error (ppm)
**Surfactin group**						
C-12 Surfactin ^#^	C_49_H_85_N_7_O_13_	5.91	980.6284	1002.6104	980.6276	−0.82
C-13 Surfactin ^#^	C_50_H_87_N_7_O_13_	6.22	994.6440	1016.6260	994.6445	0.50
C-13 Surfactin ^#^	C_51_H_89_N_7_O_13_	6.39	1008.6597	1030.6417	1008.6606	0.89
C-14 Surfactin ^#^	C_52_H_91_N_7_O_13_	6.65	1022.6753	1044.6573	1022.6745	−0.78
C-15 Surfactin ^#^	C_53_H_93_N_7_O_13_	7.11	1036.6912	1058.6732	1036.6905	−0.68
C-14 Surfactin *^,#^	C_52_H_91_N_7_O_14_	5.66	1038.6702	1060.6522	1038.6667	−3.37
C-16 Surfactin ^#^	C_54_H_95_N_7_O_13_	7.16	1050.7059	1072.6879	1050.7073	1.33
C-15 Surfactin *^,#^	C_53_H_95_N_7_O_14_	5.41	1054.7029	1076.6849	1054.7013	−1.52
C-17 Surfactin	C_55_H_97_N_7_O_13_	7.32	1064.7223	1086.7043	1064.7214	−0.85
C-19 Surfactin	C_57_H_101_N_7_O_13_	7.51	1078.7379	1100.7199	1078.7374	−0.46
**Iturin group**						
C-11 Iturin ^#^	C_45_H_70_N_12_O_14_	2.92	1015.5221	1037.5041	1015.5179	−4.14
C-13 Iturin A1 ^#^	C_48_H_74_N_12_O_14_	3.43	1043.5526	1065.5346	1043.5489	−3.55
C-14 Iturin A2 ^#^	C_49_H_76_N_12_O_14_	3.62	1057.5682	1079.5502	1057.5686	0.38
C-16 Iturin A6 ^#^	C_50_H_78_N_12_O_14_	3.96	1071.5839	1093.5659	1071.5805	−3.17
C-17 Iturin A8 ^#^	C_51_H_80_N_12_O_14_	4.14	1085.5995	1107.5815	1085.5958	−3.41
**Fengycin group**						
C-14 Fengycin A ^#^	C_70_H_106_N_12_O_20_	4.01	1435.7725	1457.7545	1435.7688	−2.58
C-16 Fengycin A *^,#^	C_72_H_110_N_12_O_19_	4.72	1447.8088	1469.7908	1447.8038	−3.45
C-15 Fengycin A ^#^	C_71_H_108_N_12_O_20_	4.29	1449.7881	1471.7701	1449.7848	−2.28
C-16 Fengycin A ^#^	C_72_H_110_N_12_O_20_	4.36	1463.8038	1485.7858	1463.8012	−1.78
C-15 Fengycin B ^#^	C_72_H_110_N_12_O_20_	4.49	1477.8194	1499.8014	1477.8159	−2.37
C-17 Fengycin D *^,#^	C_73_H_112_N_12_O_19_	5.05	1489.8576	1511.8396	1489.8540	−2.42
C-16 Fengycin B ^#^	C_74_H_114_N_12_O_20_	4.52	1491.8351	1513.8171	1491.8308	−2.88
C-17 Fengycin B ^#^	C_75_H_116_N_12_O_20_	4.67	1505.8510	1527.8490	1505.8460	−3.12

* Linear form of lipopeptides; ^#^ The lipopeptides were detected both in BPD1 crude extract and the commercial standards.

## Supplementary Materials

Supplementary materials can be accessed at: http://www.mdpi.com/1420-3049/21/12/1670/s1.
