# Supplementary material for: Clarification of the Antagonistic Effect of the Lipopeptides Produced by Bacillus amyloliquefaciens BPD1 against Pyricularia oryzae via In Situ MALDI-TOF IMS Analysis
Source: Molecules. 2016 Dec 3;21(12):1670. doi: 10.3390/molecules21121670 (PMC6273258; doi:10.3390/molecules21121670)
Supplement: Supplementary file 1 [file molecules-21-01670-s001.pdf]

Supplementary Materials: Clarification of the Antagonistic Effect of the Lipopeptides Produced by *Bacillus amyloliquefaciens* BPD1 against *Pyricularia oryzae* via In Situ MALDI-TOF IMS Analysis

Jen-Hung Liao, Pi-Yu Chen, Yu-Liang Yang, Shu-Chen Kan, Feng-Chia Hsieh, and Yung-Chang Liu

1. Antagonistic Bioassay for Ba-BPD1

1.1. Results

Antifungal and Antibacterial Activities of Ba-BPD1

Based on the results of antagonistic plate bioassay, Ba-BPD1 can antagonize 21 phytopathogenic fungi (Table S1 and Figure S1) and 12 phytopathogenic bacteria (Table S2). The antifungal and antibacterial spectra of Ba-BPD1 were wide. This indicates that Ba-BPD1 is suitable to be developed as a widely-used biocontrol agent.

Table S1. Average inhibition distance between Ba-BPD1 disc and the fungus inoculum.

Species	Disease	Average Inhibition Distance (mm)
Botrytis elliptica (Be)	lily grey mold	9.2
Botrytis cinerea (Bc)	rose grey mold	8.8
Glomerella cingulata (Gc)	mango anthracnose	3.1
Colletotrichum musae (Cm)	banana anthracnose	9.8
Rhizoctonia solani (Rs)	sheath blight of rice	4.0
Fusarium oxysporum f. sp. *Pisi* (F307)	root rot of pea	10.5
Fusarium oxysporum f. sp. *Lycopersici* (F308)	fruit rot of tomato	5.2
Fusarium oxysporum f. sp. *Lycopersici* (Fol-33)	fruit rot of tomato	7.7
Fusarium solani (FSO)	Fusarium root rot of orchid	7.3
Fusarium solani (FSL)	Fusarium root rot of litchi	7.5
Sclerotium rolfsii Saccardo (Sr)	southern blight of lily	3.0
Alternaria mali (Am)	leaf spot of apple	8.0
Phytophthora capsici (PcS1)	Phytophthora blight of sweet pepper	5.0
Aspergillus niger (An12)	Aspergillus of onion	5.0
Aspergillus niger (An22)	Aspergillus of onion	4.0
Penicillium italicum (Pi13)	blue mould of citrus	13.5
Penicillium italicum (Pi28)	blue mould of citrus	12.3
Colletotrichum gloeosporioides (Cg-T4018)	anthracnose of sweet persimmon	7.8
Colletotrichum gloeosporioides (Cg-T4044)	anthracnose of sweet persimmon	9.4
Pestalotiopsis eugeniae (Pe)	fruit rot of wax apple	7.3
Botryodiplodia theobromae (Bot)	stem end rot of mango	9.3

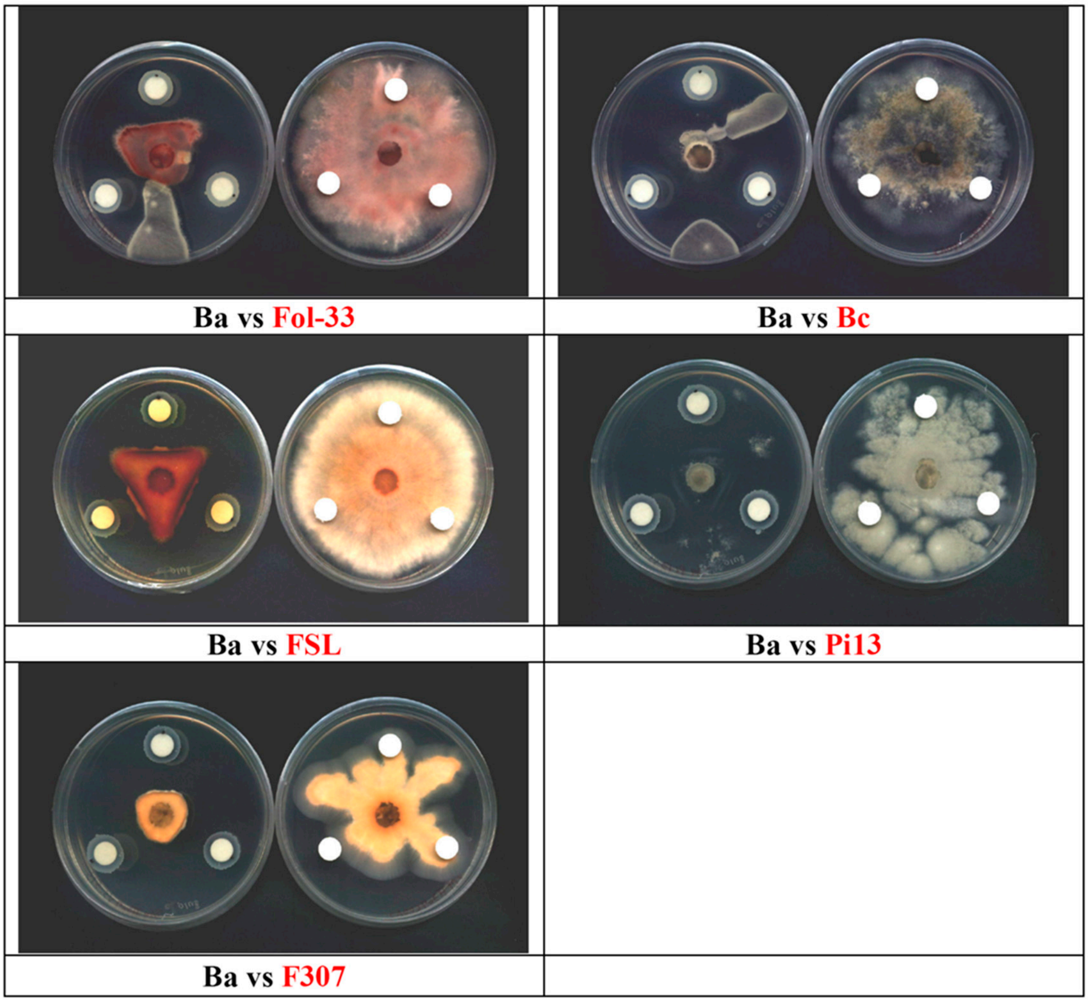

Figure S1. *Cont*.

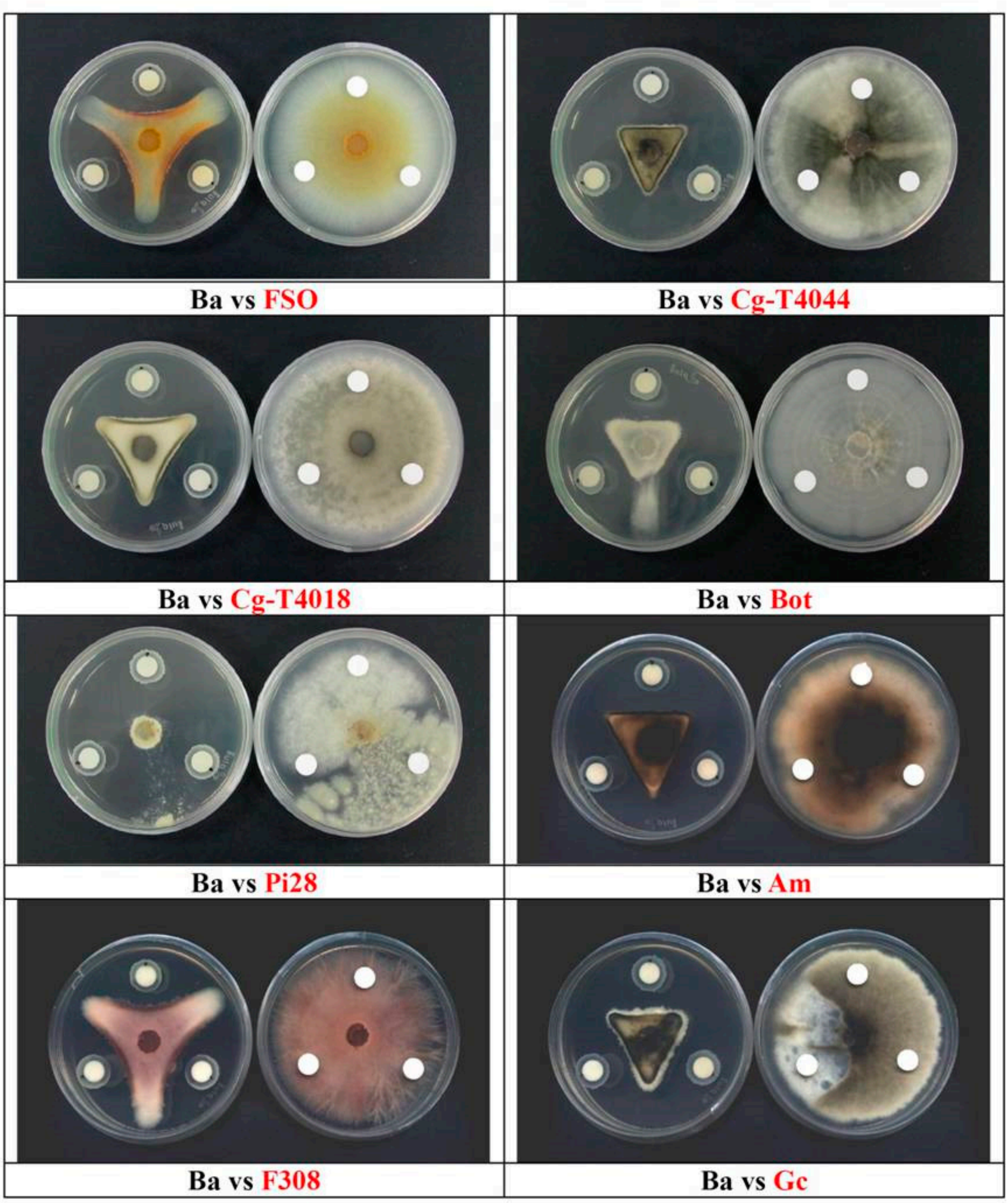

Figure S1. *Cont.*

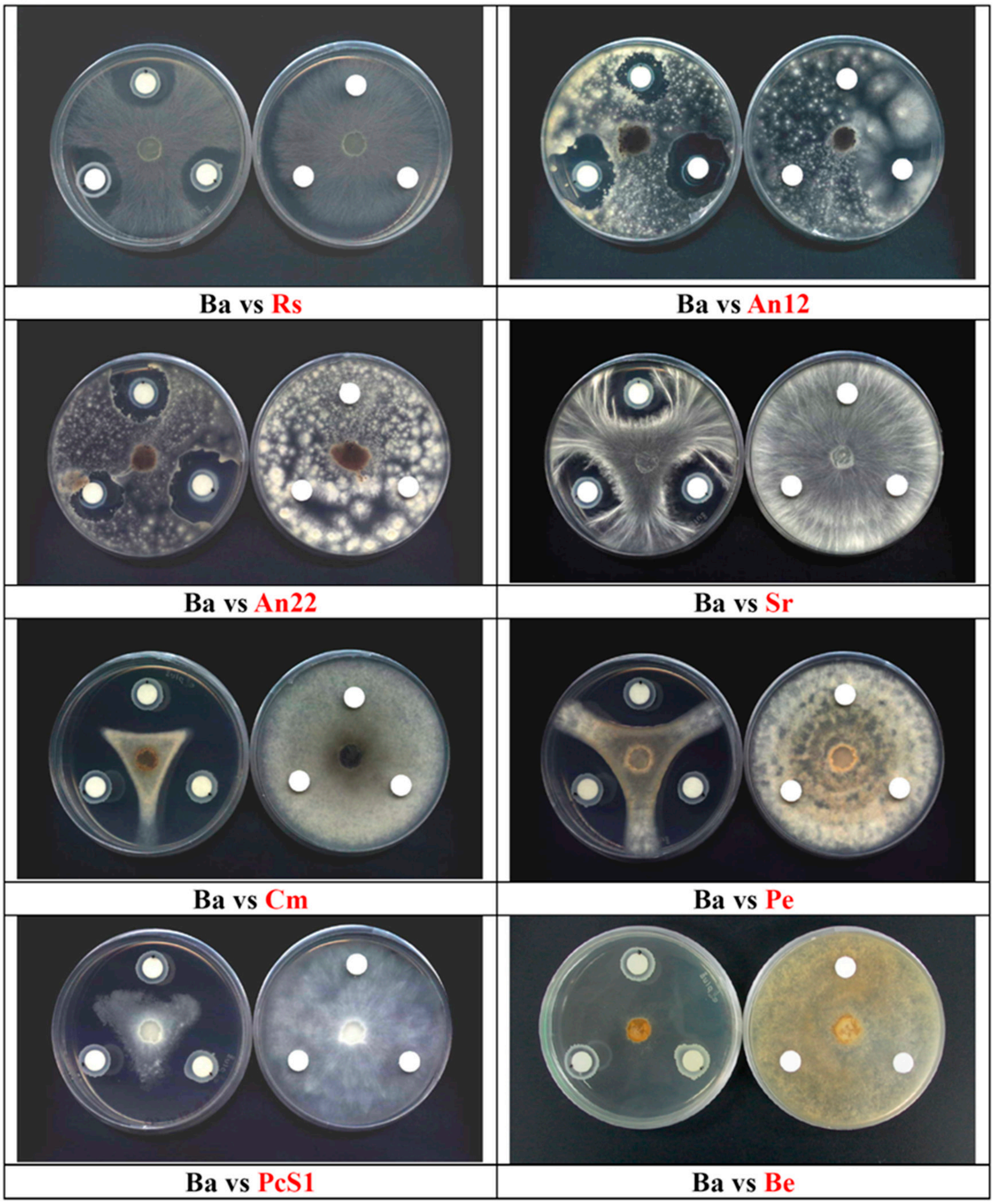

Figure S1. Antagonistic bioassay of Ba-BPD1 against phytopathogenic fungi. The short names of the fungi are listed in Table S1.

Table S2. The inhibition zone of Ba-BPD1 to the pathogenic bacteria.

Bacterium	Disease	Inhibition Zone in Diameter (cm)
Acidovorax avenae subsp. *citrulli*	Bacterial fruit blotch of melon	3.4
Agrobacterium tumefaciens	Crown gall	2.3
Burholderia caryophylli	Bacterial wilting	3.5
Enterobactor cloaceae	Bacterial basal rot	2.5
Erwinia carotovora subsp. *carotovora*	Soft rot disease	2.3
Erwinia chrysanthemi	Soft rot disease	3.1
Pseudomonas syringae	Bacterial leaf spots	3.1
Ralstonia solanacearum	Bacterial wilting	2.9
Xanthomonas axonopodis pv. *cirti*	Citrus canker	4.5
Xanthomonas axonopodis pv. *vesicatoria*	Bacterial spot of tomato	4.5
Xanthomonas compestris pv. *compestris*	Black rot of brassica	4.5
Xanthomonas oryzae pv. *Oryzae*	Bacterial leaf blight	3.2

1.2. Materials and Methods

1.2.1. Antagonistic Bioassay with Phytopathogenic Fungi

The antifungal activity of Ba-BPD1 towards 21 phytopathogenic fungi was tested on PDA plates. A plug (about 1 cm in diameter) of each phytopathogenic fungus cut from the leading edge of the cultured plate (grown on PDA at 25 °C for five days) was placed in the center of the antagonistic plate. Simultaneously, three paper discs with 9 mm diameter were placed at a distance of 1.8 cm from the fungi inoculum. Then 30 μL of Ba-BPD1 cultured broth were loaded on each paper disc. The plates were incubated at 25 °C until the fungi in untreated contrast covered the plate. The distance of the inhibition zone was measured.

1.2.2. Antagonistic Bioassay with Phytopathogenic Bacteria

The antibacterial activity of Ba-BPD1 towards 12 phytopathogenic bacteria was tested on LA plates. A paper disc with 9 mm diameter was placed at the center of plate, and 30 μL of Ba-BPD1 cultured broth (grown in LB at 30 °C overnight) was loaded on paper disc. Then the phytopathogenic bacteria-cultured broth (grown in LB at 30 °C overnight) was sprayed evenly on the LA plate. The plates were incubated at 30 °C till the inhibition zone appeared. The diameter of the inhibition zone was measured.
